# Differential response of human basophil activation markers: a multi-parameter flow cytometry approach

**DOI:** 10.1186/1476-7961-6-12

**Published:** 2008-10-16

**Authors:** Salvatore Chirumbolo, Antonio Vella, Riccardo Ortolani, Marzia De Gironcoli, Pietro Solero, Giuseppe Tridente, Paolo Bellavite

**Affiliations:** 1 Department of Morphological and Biomedical Science-University of Verona, Italy; 2 Department of Pathology-Section of Immunology-University of Verona, Italy; 3 Immunotransfusion Service-University Hospital Policlinico GB Rossi, Verona, Italy

## Abstract

**Background:**

Basophils are circulating cells involved in hypersensitivity reactions and allergy but many aspects of their activation, including the sensitivity to external triggering factors and the molecular aspects of cell responses, are still to be focused. In this context, polychromatic flow cytometry (PFC) is a proper tool to investigate basophil function, as it allows to distinguish the expression of several membrane markers upon activation in multiple experimental conditions.

**Methods:**

Cell suspensions were prepared from leukocyte buffy coat of K2-EDTA anticoagulated blood specimens; about 1500-2500 cellular events for each tested sample, gated in the lymphocyte CD45dim area and then electronically purified as HLADRnon expressing/CD123bright, were identified as basophilic cells. Basophil activation with fMLP, anti-IgE and calcium ionophore A23187 was evaluated by studying up-regulation of the indicated membrane markers with a two-laser six-color PFC protocol.

**Results:**

Following stimulation, CD63, CD13, CD45 and the ectoenzyme CD203c up-regulated their membrane expression, while CD69 did not; CD63 expression occurred immediately (within 60 sec) but only in a minority of basophils, even at optimal agonist doses (in 33% and 14% of basophils, following fMLP and anti-IgE stimulation respectively). CD203c up-regulation occurred in the whole basophil population, even in CD63non expressing cells. Dose-dependence curves revealed CD203c as a more sensitive marker than CD63, in response to fMLP but not in response to anti-IgE and to calcium ionophore.

**Conclusion:**

Use of polychromatic flow cytometry allowed efficient basophil electronic purification and identification of different behaviors of the major activation markers. The simultaneous use of two markers of activation and careful choice of activator are essential steps for reliable assessment of human basophil functions.

## Background

Human basophils, as other leukocytes, express several cell membrane antigens which can be related to their immunological responsiveness. Challenging basophils with allergens or agonists may result in a modified expression of these molecules on cell membrane, a mechanism that can be evaluated by flow cytometry [1-5]. Furthermore, the expression of membrane molecules could change also while cells are responding to a pathology state [6-9] and/or following a therapeutical treatment [10].

While most authors recognize the flow cytometric approach as a proper tool to investigate basophil function, several problems and methodological issues are still to be clarified, namely subject responsiveness (a broad variability in basophil activity is evidenced between different donors and different markers within the same donor), sample treatment (which may affect cell activation state and response pattern), gating procedure (which is a key factor to separate selectively a small population like basophils) and the selection of the best suitable activation markers [11-13]. The discovery of new monoclonal antibodies about membrane antigens has improved strategies to analyze the basophil function [2,14-17]. However, to the best of our knowledge, cytometry applied to basophil activation study is currently restricted to a two-three color measurement and to two light-scattered parameters [13,18-20].

It is conceivable that the use, in the same analytical setting, of more than two or three flow cytometry markers to evaluate the behavior of several activation molecules may render more informative the whole assay system [21]. Critical points, however, have raised a debate about the actual cost-effectiveness of an analytical strategy using more than three colors [5,21,22]. In this study we focused on the kinetics of activation markers under different cell conditions; to this purpose we needed a clear-cut distinction between phenotype and activation markers. Our protocol involved two steps. First, an electronic capture of basophil leukocytes as low side-scattered cells in the CD45^expressing ^lymphocyte area: the use of CD45 contributes to discriminate basophil area from other leukocytes excluding cellular debris [23]. Second, inside this area the electronic capture of HLA-DR^non expressing^/CD123^bright ^cells allows to identify pure basophils [24]. Although CD203c is considered a selective marker for basophils [25] this molecule is expressed at a low level in non-activated cells: the use of such a weak marker as a phenotype tracer might result into the exclusion of resting cells having a very low CD203c expression from gating capture. An essential step of this strategy is a clear-cut and quantitative evaluation of the membrane molecule changes associated with cell activation. In particular we stressed on the differential behavior of the main activation markers CD63 and CD203c in the same experimental sample. Cellular responses to different agonists were followed by evaluating the behavior of the activation markers CD63, CD203c, CD69 or CD13 compared to a resting state.

As working cell preparation, in this protocol, we used basophil-enriched buffy coats, pooled from healthy blood donors, in order to reduce possible effect of individual sensitivity, to wash out plasma which could interfere with anti-IgE activation, and to eliminate platelets which share some activation markers with basophil (CD63) [5]. We neither use Ficoll nor Percoll gradients in order to prevent spontaneous activation of these cells and to keep as close as possible a standard blood environment condition. Moreover, we did not use IgE-labeling, since anti-IgE was used as stimulatory agent, other leukocytes can be targeted by anti-IgE and because FcåRI expression varies considerably from one subject to another [4,11,26].

The choice of a multi-parametric approach allows to focus onto the differential responsiveness of several markers simultaneously and in the same sample even by the application of correlated logical gates. This approach should give more insight about the functional relationship between activation molecules, about the optimal and threshold doses for detecting cell activation, and could facilitate subsequent studies about the role of these different antigens as predictive diagnostic markers [17,27].

## Materials and methods

### Reagents and disposable ware

All reagents were prepared by using pure compounds in a laminar flow hood and with disposable plastic ware. HEPES ([4-(2-hydroxyethylpiperazine-1-ethanesulonic acid], sodium-heparine 170 U/mg and salts were purchased from Sigma-Aldrich, GmbH, Germany. Goat anti-human monoclonal IgE was from Caltag, USA; N-formyl-L-methionyl-L-leucyl-L-phenylalanine (fMLP), mouse monoclonal anti-human-IgE (clone GE), 4-brome-calcium ionophore A23187 and dimethylsulfoxide (DMSO) were purchased from Sigma-Aldrich GmbH, Germany. Anti-CD203c-PE (isotype IgG_1_, clone 97A6) was purchased from Beckman Coulter Immunotech, USA, anti-CD69-APC (isotype IgG_1_, clone FN50), anti-CD123-PECy5 (isotype IgG_1_, clone 9F5), anti-CD13-APC (isotype IgG_1_, clone WM15), anti-HLA-DR-PECy7 (isotype IgG2a, clone L243), anti-CD45-APCCy7 (isotype IgG_1_, clone 2D1) and anti-CD63-FITC (isotype IgG_1_, clone H5C6) were purchased from Becton Dickinson Pharmingen USA. Vials and test tubes were purchased from (BD Falcon, NJ, USA).

### Basophil preparation

Basophils were collected by pooling the leukocyte buffy coats drawn from venous K_2_-ethylen-diamino-tetra-acetic acid (EDTA) anticoagulated peripheral blood of at least four healthy, non allergic, screened subjects (blood donors) for each experiment [20]. Blood samples were worked out within 2–3 hours from venous withdrawal using a differential centrifugation step procedure. Blood specimens were diluted 1:4 into an ice-kept or refrigerated (+2°C/+8°C) HEPES modified buffer ([4-(2-hydroxyethylpiperazine-1-ethanesulonic acid] 20 mmol/L; NaCl 127 mmol/L; KCl 5 mmol/L; sodium-heparin 5 UI/ml, pH 7.4) using sterile 14-ml polypropylene cap-equipped round bottom tubes and centrifuged at 700 *g *for 15 minutes at +4°C. Leukocyte buffy-coat layers, contaminated by erythrocytes, were individually collected, suspended in the HEPES-heparin (HBE) buffered solution and centrifuged at 400 *g *for 10 minutes at +4°C. Pelletted buffy-coats were washed out from surnatants, collected in a single polypropylene tube, suspended in the ice-cold HBE medium and centrifuged at 400 *g *for further 10 minutes. Finally pooled leukocyte buffy-coats were suspended to 1:4 v/v compared to whole blood starting volume with the refrigerated HBE buffer. In order to maintain basophils in a resting state during the whole preparation procedure, cells were treated with apyrogenic solutions and sterile disposable plastic ware and kept into ice until use in order to prevent any spontaneous activation [17]. An aliquot of about 1 ml of the above HBE-suspended cell culture was transferred to a Bayer ADVIA 2120 automated hematocytometer for basophil counting and yield [28].

### Cell treatment

fMLP was dissolved in dimethylsulfoxide (DMSO) as a 2 × 10^-2 ^M stock solution, stored at -20°C and thawed before use. Each solution was freshly prepared by diluting fMLP in HBE supplemented with 5 mM CaCl_2 _and with 2 mM MgCl_2 _(HBC buffer) to 2× the final concentration, in order to make the indicated working solutions. Anti-human monoclonal IgE, purchased as mouse ascitic fluid with a concentration of 7.3 mg/ml (4.87 × 10^-5 ^moles/L), or as purified goat-anti human (0.5 mg/ml) in buffered saline with NaN_3_, was diluted in HBC buffer two times the final molarity, in order to make the indicated working concentrations. Calcium ionophore A23187 was dissolved in DMSO at the stock concentration of 10^-3 ^mol/L and then dissolved in HBC buffer to 2× the final concentration, in order to make the indicated working solutions.

Ten minutes before incubation, the cell suspension was diluted 1:1 v/v with HBE and brought at 37°C; dilution was carried out to reduce homotypic aggregation following cell activation [29]. Stimulation with agonists was performed at 37°C for the indicated time inside round bottom, cap-equipped polystyrene 5-ml (12 × 75 mm) plastic test tubes. 50 μl of agonist or of control HBC for resting samples were distributed in the test tubes and brought to 37°C. Then 50 μl of the cellular buffered suspension were added and incubation carried on for the indicated time. Cell culture homogeneity was maintained by gentle mixing test tubes every ten minutes. The incubation was then stopped by adding 100 μl of ice-cold HBE buffer supplemented with 2.8 mmol/L Na_3_-EDTA and samples were put on ice until staining with monoclonal antibodies.

### Staining with monoclonal antibodies

In order to choose the most suitable fluorochromes for antibodies, we followed previously reported settings [30]. The protocol included tandem-dyes for cell phenotyping and small organic molecules or proteins (FITC, PE and APC) for the activation markers and used a 488 nm-633 nm two lasers equipped air flow cytometer (Becton Dickinson FACScanto). For tandem-dyes stability we followed manufacturer's instructions. Staining protocols were performed at 4°C for 20 minutes following manufacturer's instructions and according to this pattern: 10 μl/200 μl cell suspension for all the antibodies except for the following markers CD45-APCCy7 and HLA-DR-PECy7 (5 μl/200 μl cell suspension).

### Preparation of flow cytometry samples

Soon after staining, samples underwent erythrocyte lysis according Tsang's protocol [31]: lysis was performed with 3 ml of a +4°C refrigerated ammonium-chloride solution (NH_4_Cl 155 mmol/L; Na_2_HCO_3 _10 mmol/L, Na_3_EDTA 0,10 mmol/L, pH = 7,2) for 2 minutes on ice, then cells were pelleted at 500 *g *for 5 minutes in a refrigerated centrifuge. Surnatants were removed and pellets gently resuspended in 0.5 ml of a BD-Isoflow phoshpate saline (PBS) balanced buffer, just ready for flow cytometry assay.

### Gating procedure

Basophils were gated as low side-scattered cells (SSC) in the CD45^dim ^lymphocyte area: the use of CD45 contributed to discriminate basophil area from other leukocytes better than FSC/SSC light-scattering, excluding cellular debris and to account for a further selective marker to phenotype basophilic cells [23]. This region was investigated for HLADR and CD123 expression. Cellular events with a HLADR^non expressing^/CD123^bright ^phenotype were then identified as basophils [24,32]. For each sample about 50,000 events were acquired in which, by applying this gating protocol, approximately 1500–2500 basophils in the gate were counted.

### CD63 intracellular evaluation

To evaluate intracellular CD63, basophils were stained as the above described methods excluding CD63-FITC. Then the cells underwent a lytic treatment, were fixed with 4% paraformaldehyde and permeabilized with 0.1% saponin and 0.09% sodium azide phosphate balanced modified buffer (BD CelLyse buffer) and finally stained with CD63-FITC as above. Samples were reconstituted in BD-Isoflow PBS balanced buffer and read.

### Cytometer and fluorochrome setting

Flow analysis was performed using a two laser BD FACScanto flow cytometer: this instrument has a 10.000 events/sec capability, six-color detection and 0,1% of sample carryover. Analyses were performed with a mean flow rate of 300–500 events/sec, setting an excess limit of 50,000 events to record in the basophil gate in order to evaluate the whole buffered suspension volume and having a proper estimation of cell recovery and reproducibility. Compensation followed cytometer manufacturer's instruction according an off-line procedure by applying automated electronics algorithms and preset templates, by using bi-parametric logarithmic dot plots, gate-specific tubes and single-tube data analysis, and optimizing FSC threshold and fluorochrome voltage as set up parameters.

To evaluate fluorochrome unspecific staining, isotype controls for anti-IgG_1 _and anti-IgG_2α _were introduced in the preliminary procedure to set up photomultiplier and instrument technical parameters; this control used a staining procedure carried out without introducing in the assay system the fluorochrome of interest was also performed.

### Sample analysis and data collection

Mean of fluorescence intensity (MFI) for each fluorochrome-labeled monoclonal antibody was calculated automatically with the cytometer software by averaging the total fluorescence of the marker in the basophil gate. As well percentage of activated cells was calculated by the software considering the CD63-FITC bright cells counted to the right of a threshold that was established including the main peak of fluorescence of a sample of resting cells. In order to reduce standard deviation due to brightly fluorescent cells respect to dimly ones, a logarithmic scale and the coefficient of variation to measure variability dispersion were used. When necessary, Bigos' formula to normalize brightness over background was applied [22].

## Results

### Basophils yield and electronic sorting

The samples from a total of 82 healthy subjects (38 males, 44 females, mean age 46.34 yrs ± 5.67 SD, range 26 to 65 yrs) were used, in a total of 21 experiments. Starting from K_2_-EDTA anticoagulated peripheral blood with a WBC ×10^3^/μl mean count of 6.41 ± 0.90 SD, corresponding to a basophil concentration mean of 37.87 ± 10.70 SD cells/μl, pooled buffy coats, having a mean count of 8.47 ± 2.09 SD WBCx10^3^/μl and an estimated basophil concentration of 92.25 ± 18.43 SD cells/μl, were obtained. This yield corresponds to an enrichment that is approximately 2.4 times in respect to starting whole blood.

**Figure 1:**
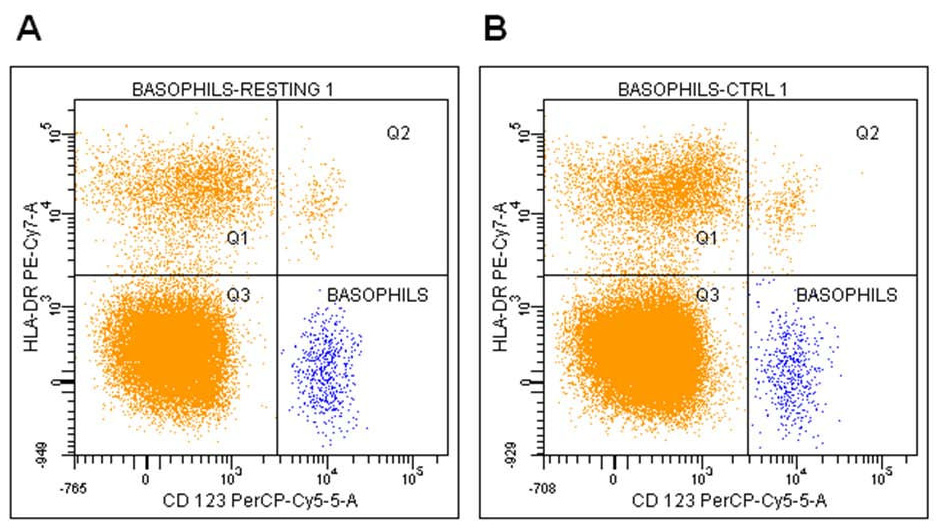
**Human basophils gating with the BD-FACScanto flow cytometer**. Leukocytes are immunologically gated in the CD45^dim^-lymphocyte area and electronically captured as cellular events in HLADR^non expressing^/CD123^bright ^biparametric logarithmic plot to distinguish them from other events and identified as basophils from monocytes (Q1), plasmacytoid dendritic cells (Q2) and lymphocytes (Q3). (A) Resting cells, (B) activation with 1 μg/ml goat anti-human IgE.

Figure 1 shows a typical dot plot of basophil immunologic phenotyping and electronic capture. HLADR-PECy7/CD123-PECy5 plot, built using the CD45-dim cells in the lymphocyte area (see methods), allowed the definition of a well-isolated area of CD123^bright ^cells, not expressing HLA-DR (basophils) (Figure 1A) [32]. This electronic cloud did not change its position upon activation with any agonist (here the result with anti-IgE is shown, Figure 1B, see also Table 1 [see Additional file 1]). This last point is particularly important in studies designed to investigate the dynamics of activation markers compared to a basal, resting state.

### Mean fluorescence markers in the population

Table 1 [see Additional file 1] summarizes MFI results concerning a series of experiments performed in our laboratory. The data show the changes induced by the two agonists employed on the different markers. Gating markers (CD123 and HLADR) did not significantly change their membrane expression under activation, while CD45 was up-regulated by 2–3 times. Such an MFI increment extent was also observed with CD203c triggered by fMLP or by anti-IgE and with CD13 triggered with fMLP. CD63-FITC MFI, very low in resting cells (mean 619.76), increased by 24 times following fMLP and by 4.8 times following anti-human IgE stimulation. The mean percentage of CD63-FITC^bright ^cells increased from 3.12% to 33.44% following fMLP activation and from 3.12% to about 13.67% following anti-IgE stimulation. The N-aminopeptidase CD13 and the c-type lectin CD69 did not show, however, a reproducible activation pattern in our assay condition, which led us to focus on CD63 (*lysosome-associated membrane protein-3 *or LAMP-3) and CD203c (*ecto-nucleotide-pyrophoshatase phosphodiesterase-3 *or ENPP-3) markers hereafter.

### Basophil activation markers: CD63 and CD203c

Basophil response towards the different agonists was examined following changes in the mean fluorescence intensity (MFI) associated to specific membrane marker fluorochrome and by evaluating dot plots of the acquired events.

**Figure 2:**
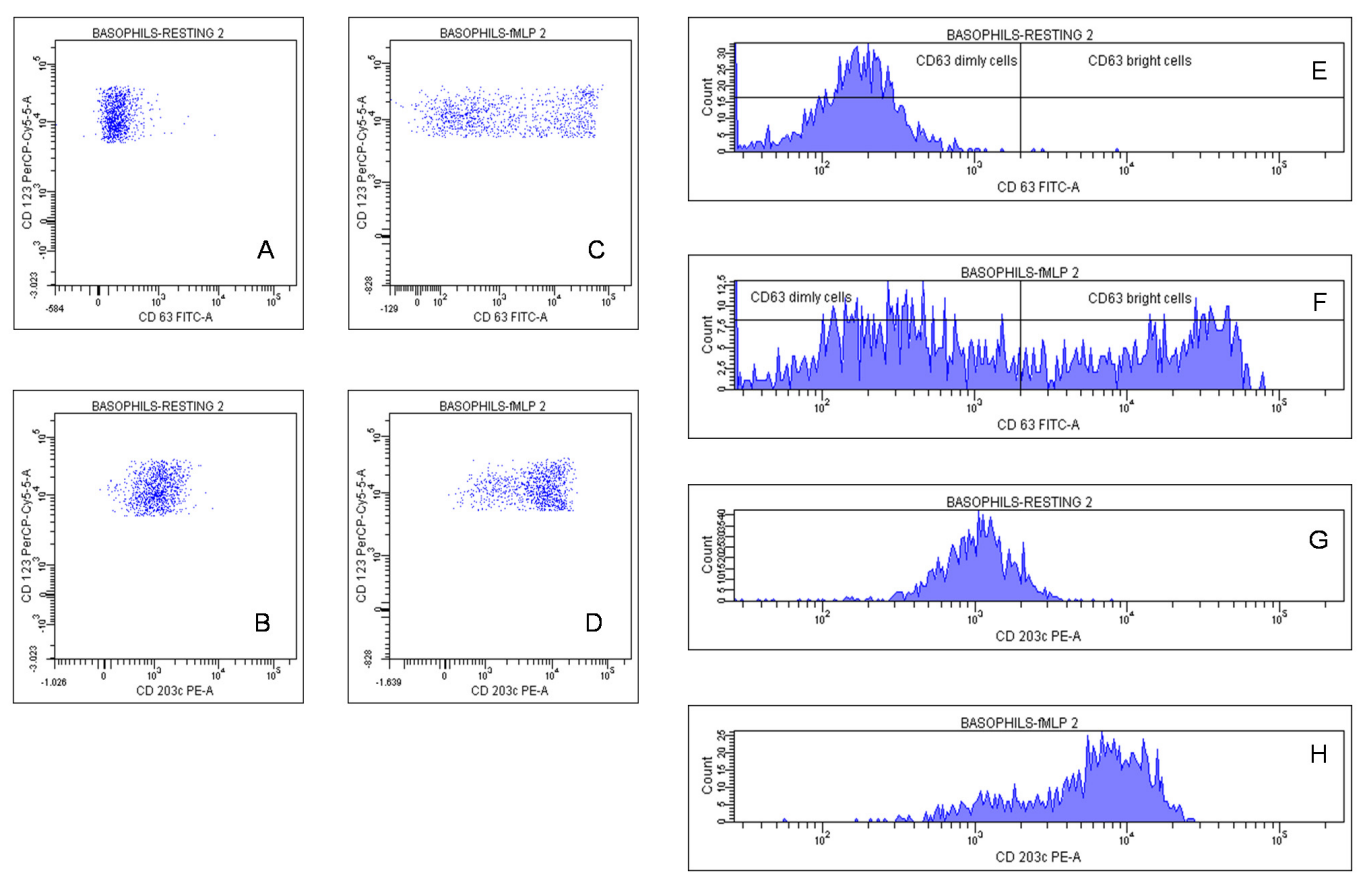
**Dot plots and histograms of CD63 and CD203c expression in response to fMLP**. Basophils were incubated in the absence (A,B,E,G) and in the presence (C,D,F,H) of 10^-7 ^M fMLP at 37°C for 30 minutes and gated basophils were plotted both as dot plots (A,B,C,D) and as histograms (E,F,G,H).

**Figure 3:**
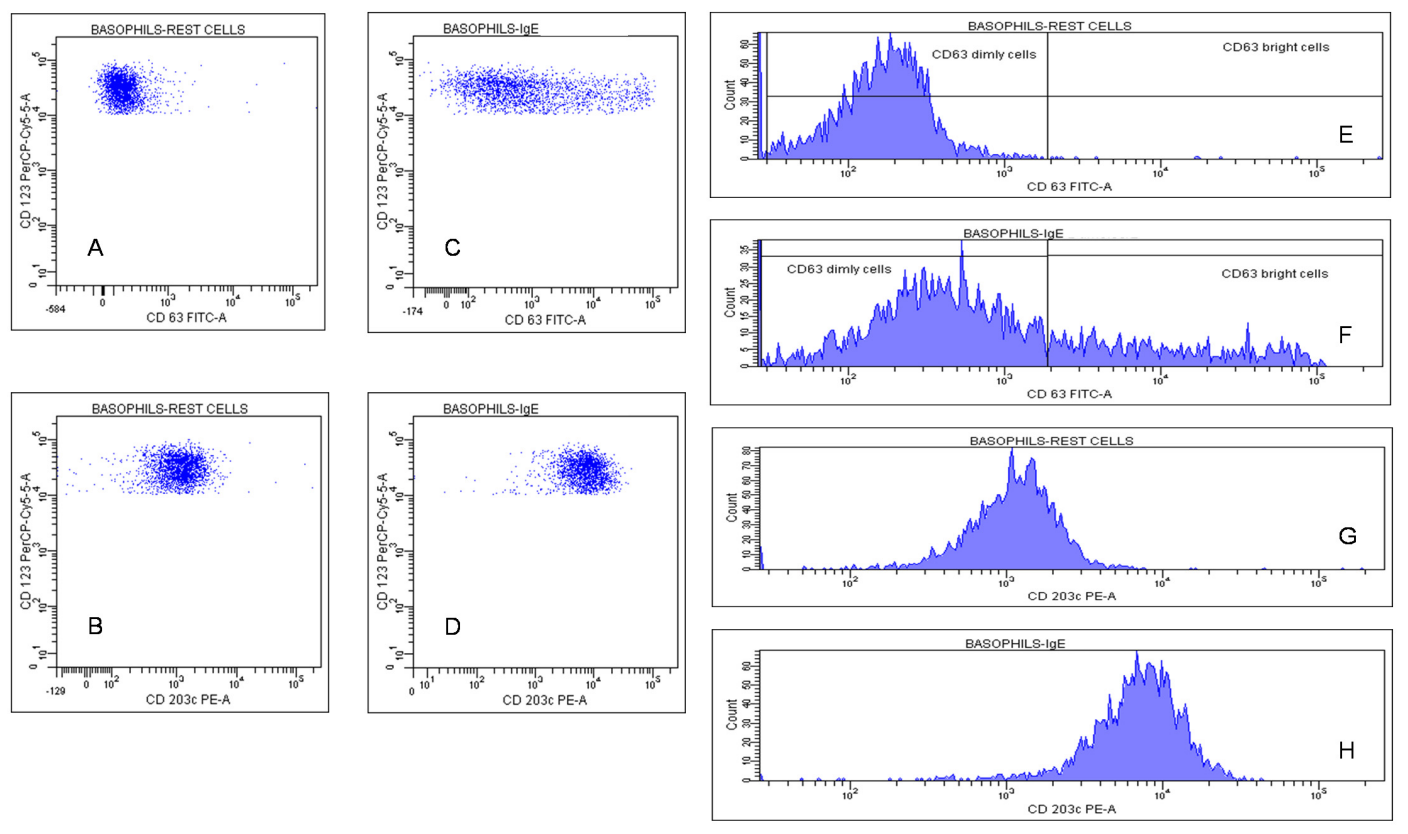
**Dot plots and histograms of CD63 and CD203c expression in response to anti-IgE**. Basophils were incubated in the absence (A,B,E,G) and in the presence (C,D,F,H) of 10 μg/ml goat anti-IgE at 37°C for 30 minutes and gated basophils were plotted both as dot plots (A,B,C,D) and as histograms (E,F,G,H).

Figures 2 and 3 show a typical experiment of cell activation. Non activated basal (resting) basophils behaved as typical non expressing cells for CD63 but expressing CD203c, although at a low level (~3000 fluorescence units) (Figure 2A and 2B): fluorescence histograms (Figure 2E and 2G) exhibited a normally distributed population of both markers. Following 30 minutes of incubation with 100 nM fMLP, a significant fraction of cells (41.4%) showed a CD63 bright phenotype (Figure 2C and 2F), while almost all basophils up-regulated CD203c membrane expression (Figure 2D and 2H). The difference between CD63 and CD203c expression following cell activation was even more striking using anti-human IgE as stimulatory agent in a simultaneous assay of the same cell preparation (Figure 3). Basal (resting) level of CD63 (Figure 3A) and of CD203c (Figure 3B) and their respective fluorescence distribution (Figure 3E and 3G) were comparable to those of the resting cells previously described. Following incubation with 10 μg/ml anti-IgE, CD63 membrane expression occurred with a lesser extent than with fMLP triggering and at only 22.4% of the cells (Figure 3C and 3F), while the CD203c expression increased in all basophils and at the same extent as with fMLP triggering (Figure 3D and 3H).

**Figure 4:**
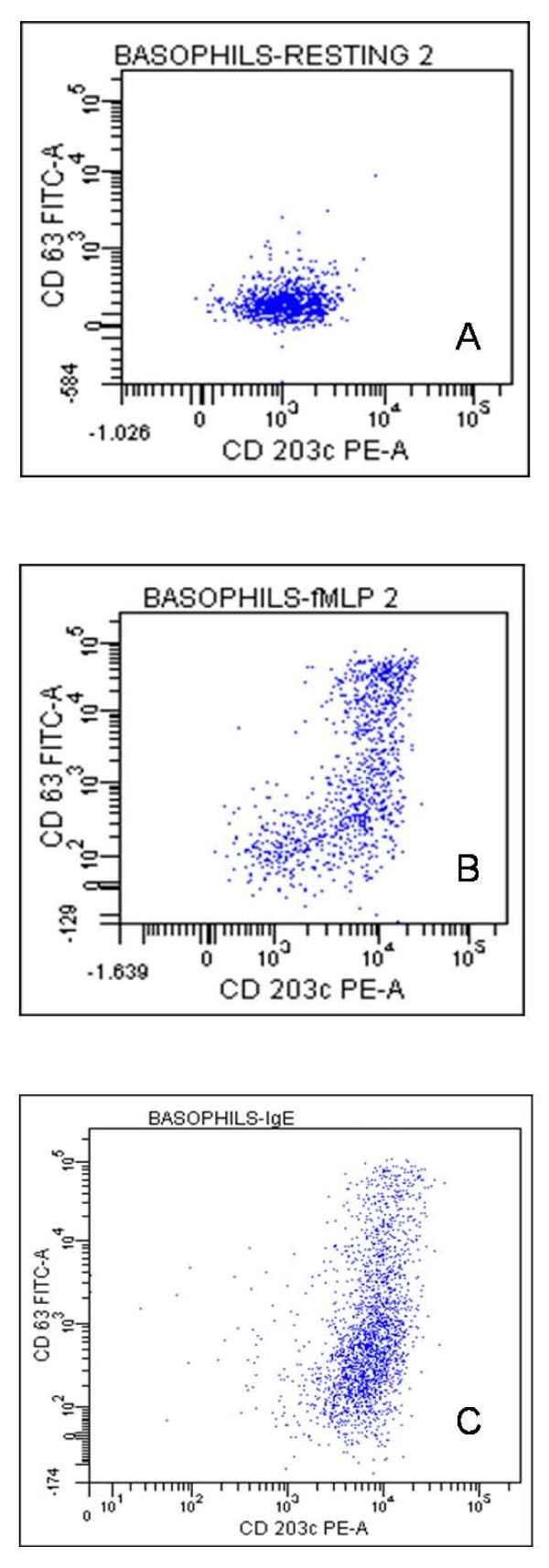
**Biparametric dot plots of CD63 and CD203c expression**. Biparametric dot plots showing CD63 and CD203c in resting basophils (A) and after activation with 100 nM fMLP (B) or with 10 μg/ml goat anti-IgE (C).

**Figure 5:**
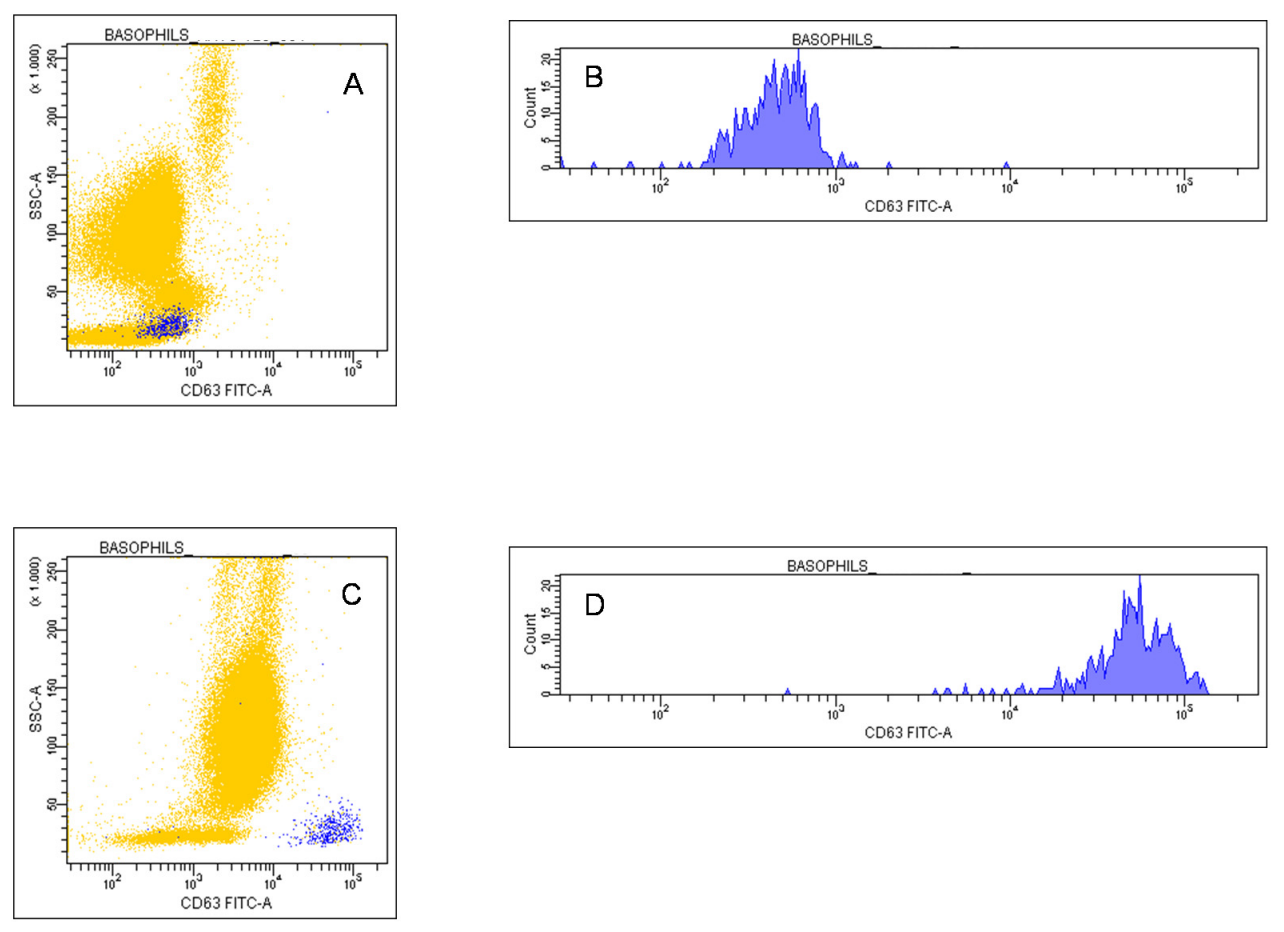
**Intracellular CD63**. Dot plots (A,C) and histograms (B,D) of CD63-associated fluorescence in normal (A,B) and saponin-permeabilized human basophils (C,D). See text for methods.

These two major markers were examined in the same biparametric dot plot (Figure 4). Resting (CD63 negative) basophils showed a significant CD203c-fluorescence on their membrane (Figure 4A), as expected. Following fMLP (Figure 4B) and anti-human IgE (Figure 4C) stimulation, basophils evidenced complex activation patterns, in which a population of basophils expressing a CD63^dim^/CD203c^bright ^phenotype is clearly evident. This might indicate: a) the presence of a CD203c^bright ^basophil sub-population lacking CD63 tetraspanin or b) that the CD63 dimly cells have this intracellular-associated protein 3 but are unable to up-regulate it on the membrane. To examine these issues, we investigated intracellular storage of CD63. Figure 5 shows that all basophilic cells, having a CD63^non expressing ^phenotype on their membrane (Figure 5A and 5B), have a CD63^expressing ^phenotype with intracellular staining (Figure 5C and 5D). This suggests that CD63 is present in all the cells but is upregulated (expressed on the cell membrane) only in a few of them.

### Dose response and time course

**Figure 6:**
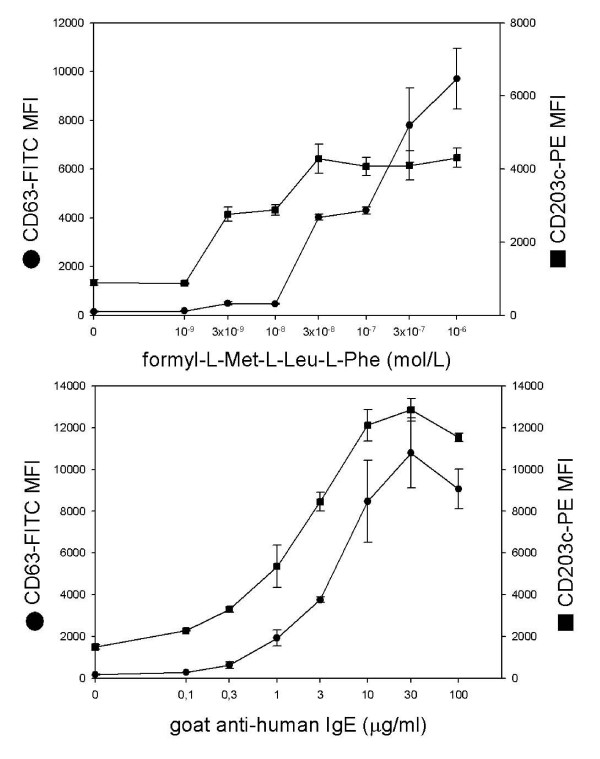
**fMLP and anti-IgE dose response of CD63 and CD203c expression**. fMLP (a) and goat anti-human IgE dose response (b) of the activation markers CD63 and CD203c in a typical triplicate experiment of four performed. Basophils were incubated for 30 minutes at 37°C.

To evaluate the responsiveness of basophilic cells in order to assess their normal function in our testing condition, dose response and time course were performed. In fMLP dose-response curves, CD203c appears the earlier marker to be activated by formylated peptides: at a dose of 3 × 10^-9 ^M, CD203c MFI increased from 873 to 2765 (growing up to three times) while CD63 reached the maximum of activation at 3 × 10^-8 ^M (changing MFI from 179 to 4020). Moreover, while CD63 up-regulation seems to go forward up to 10^-6 ^M fMLP, CD203c reached a maximum plateau at the dose in which CD63 began to increase (Figure 6A). On the other hand, with the agonist anti-IgE the two curves were parallel and there was no dissociation between the two markers (Figure 6B).

**Figure 7:**
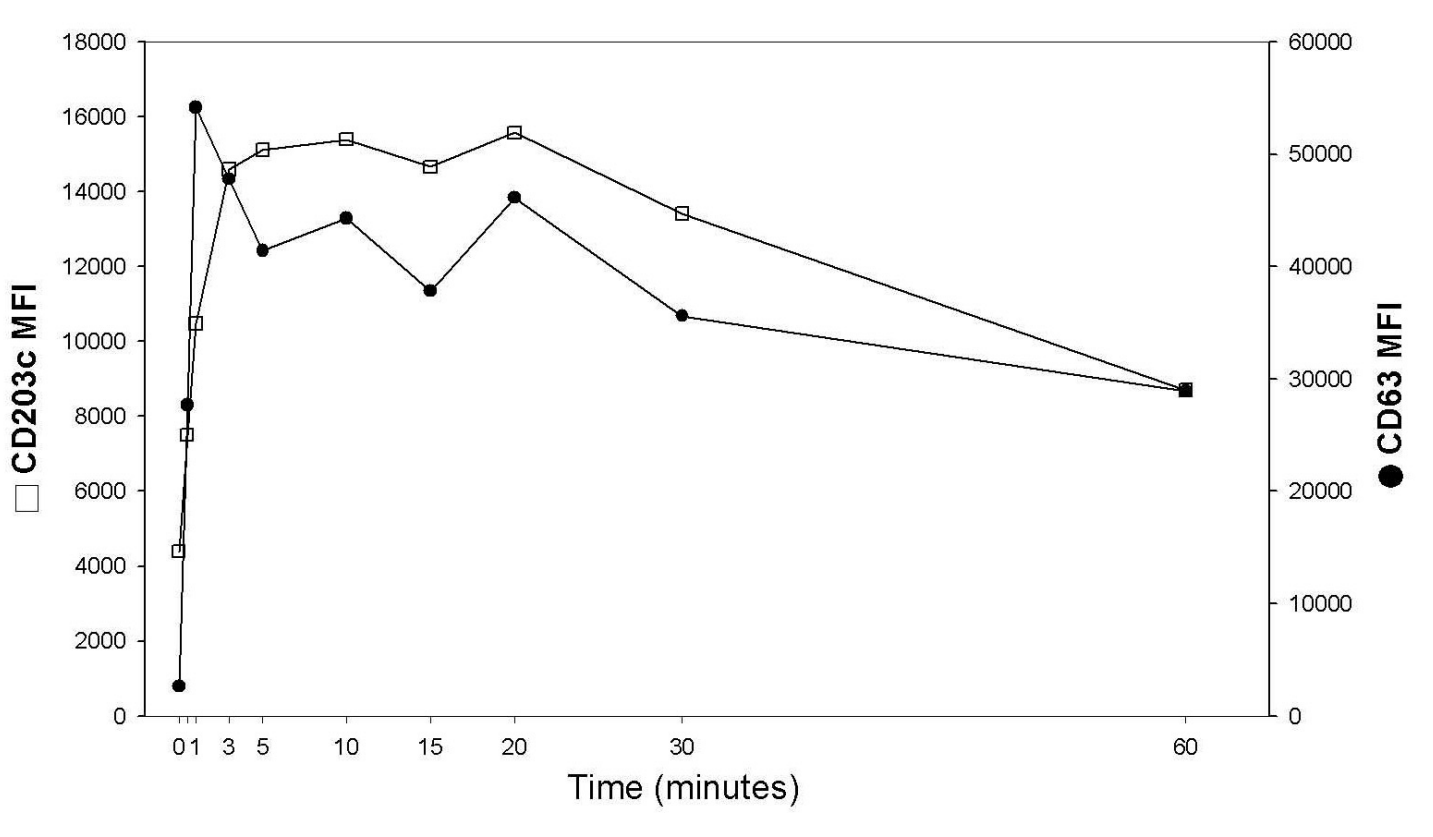
**Time course of CD63 and CD203c expression**. Basophils were incubated with 10^-7 ^M fMLP at 37°C and activation stopped with cold HEPES-buffered solution containing 2.8 mM Na_2_-EDTA.

Time-related response patterns were investigated using an optimal stimulatory dose of formylated peptides (Figure 7). CD63 up-regulation occurred within the first minute of activation while CD203c membrane reached the plateau after about three minutes: the expression of both markers was slightly down-regulated after 60 minutes following agonist treatment. A 15 to 30 minutes time appears as optimal for the full expression of cell activation using those markers.

**Figure 8:**
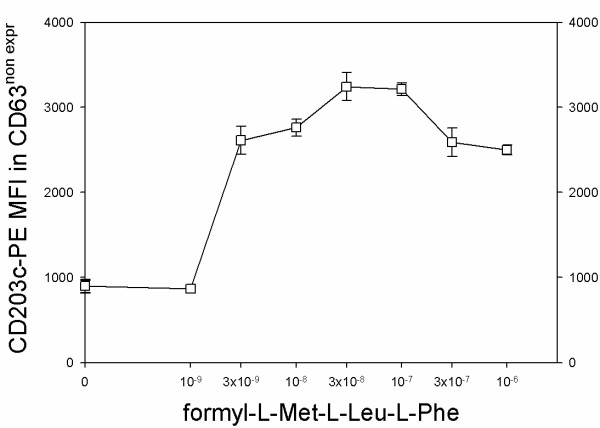
**Expression of CD203c in CD63^non expressing ^human basophils**. Basophils were incubated for 30 minutes at 37°C in the presence of different concentrations of fMLP and the MFI of CD203c was evaluated in the subset of CD63^non expressing ^basophils.

In order to better describe the dissociation of two markers observed with fMLP as agonist, the response of CD203c in CD63^non expressing ^basophils was evaluated (Figure 8). CD203c dose response in the basophilic cell population which did not express CD63 basophils was comparable to that evaluated by taking into account all the basophils (see also Figure 6A), indicating that sensitivity to fMLP did not depend on the differential CD63 expressing subpopulation.

**Figure 9:**
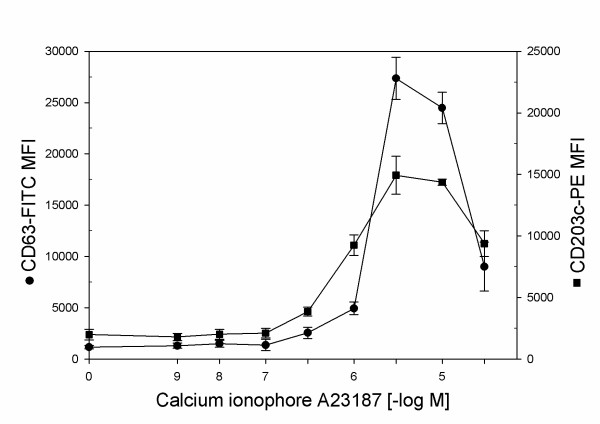
**Dose response curves of CD63 and CD203c following stimulation with A23187**. Basophils were incubated for 30 minutes at 37°C in the presence of increasing doses of the calcium ionophore A23187.

To investigate a possible dissociation of two activation markers at the level of the intracellular signaling, we examined CD63 and CD203c dose response to the calcium ionophore A23187. CD203c response to A23187 was similar to the response exhibited by CD63, both markers reaching a peak close to 3 × 10^-6 ^M A23187 (Figure 9).

## Discussion

In this work human basophil activation was investigated in vitro by using a two laser multiparameter flow cytometry, a technique that is becoming prevalent in immunophenotyping. The latter was applied to leukocyte preparations from buffy-coats of human blood samples, yielding basophil enriched populations, with cell numbers sufficient to perform several activation studies under different experimental conditions. As starting material, small (3 ml) samples of K_2_-EDTA-anticoagulated blood were employed and allowed the preparation of cells that proved to be in a basal, resting state and to be highly responsive to *in vitro *stimulation. Besides providing enriched and functional leukocyte samples, the overall procedure of basophil isolation and pooling has the advantages of washing out the plasma and the majority of platelets and of reducing the impact of the great variability of individual responsiveness [33]. This may be particularly important for *in vitro *testing of agonists and drugs.

Basophil electronic capture was very efficient by using a HLADR^non expressing^/CD123^bright ^gating strategy. Although CD203c is a selective marker for basophilic cells, we did not choose this molecule as phenotyping tracer since the brighter CD123 revealed as a better marker to separate cells and because we used the CD203c for activation studies.

Basophils appeared as highly responsive to agonist triggering in a time-course and dose-response manner. We explored several classic markers that were previously described in the literature [9,14,34-36], with the aim to investigate their expression in our experimental system, their possible advantages and features.

After basophil treatment with fMLP and with anti-IgE, an increase in MFI of CD63, CD203c, CD45 and CD13 was observed (the latter only with fMLP), while CD69, used by others as activation marker [1,17,37-39], did not show appreciable changes in these conditions. This discrepancy with our data may be due to the fact that the cited protocols using CD69 introduce interleukin-3 as priming agent [40]. Among the five activation markers tested, the most sensitive, reproducible and evident differences with respect to the resting state were noted using CD63 and CD203c. However, these two markers behave in a quite different way: cells "non responding" to fMLP or anti-IgE (when evaluated using CD63) are fully responsive to the same agonists when evaluated using CD203c. This observation may have clinical and laboratory importance since in the literature the existence of "non responder" individuals in basophil activation tests has been reported [12,41]. If only CD203c is able to trigger full functional activation of basophils, the identification and diagnosis of the so-called "non responder" individuals in clinical laboratory analysis should be performed by employing at least two markers of activation and not the single CD63. In our experimental setting very low doses of fMLP or anti-IgE triggered a clear-cut CD63 and CD203c expression when we started from a fully resting condition. Due to its sensitivity, this approach may be suitable to evaluate this pattern also in drug hypersensitivity and allergy diagnosis. In dose response curves of fMLP, the full expression of CD203c precedes by one-two orders of magnitude the full expression of CD63. This indicates that low doses of the bacterial peptide mobilize one or more intracellular transduction pathways that are fully competent for triggering CD203c, but not CD63. This observation may have a physiological meaning, as CD203c is an ecto-nucleotidase, belonging to the nucleotide pyrophoshatase/phosphodiesterase family, that may be useful on the basophil membrane for other, subtle, regulatory changes, possibly related to purinergic signaling [42-44]. Alternatively, the ectoenzyme may be involved in the preparation (priming) to degranulation events. However, this hypothesis does not fit with the time-course experiment where, at optimal fMLP doses, the CD63 expression (degranulation) peaks at 1 min while CD230c requires 3–5 minutes to optimal expression.

With anti-IgE and calcium ionophore as agonists, the two markers behaved in a similar way in the dose-response curve, suggesting that the discrepancy between the two markers, noted with fMLP, is receptor-specific and does not belong to a calcium-activated pathway. It is tempting to speculate that fMLP, being both a chemotactic factor and a degranulating agonist, requires some specific regulation, which may be more sophisticated and flexible than those required by anti-IgE, the latter being a substance that mimics the physiologic way of basophil activation that induces the histamine release. Interestingly, in human neutrophils intracellular calcium release and actin polymerization require fMLP doses much lower than those necessary for superoxide production activation and lysozyme release [45,46], suggesting the existence of dual pathways of regulation in both leukocyte types. This indicates that all the cells have normal fMLP receptors and are functional; moreover, all the cells are positive upon intracellular staining of CD63. Therefore, our data suggest the existence of a main subpopulation of fully viable and responsive, but non degranulating basophils in human blood, and this is in agreement with other, independent, observations [47,48].

## Conclusion

In summary the results from this multiparametric approach suggest:

a) CD63 is the most sensitive and early activation marker of human basophils, in agreement with previous evidence from literature. However, CD63 is expressed in only a minority of basophils even at optimal agonist doses, while it is present in all the cells as intracellular pool.

b) CD203c is less sensitive as activation marker, because there is a substantial expression also in the resting state. On the other hand, this marker is upregulated in all the cells following activation, also in those that are not identified as activated using CD63.

c) The dose-response curves show a receptor-specific (observed with fMLP but not with anti-IgE and calcium ionophore) dissociation between the two activation markers. This evidence suggests that the molecule marked by CD203c is associated with the low-dose events of chemotaxis while the molecule CD63 is associated with degranulation, as previously reported.

d) An overall view of several activation molecules within the same experiment, using pooled cell preparations could be an affordable tool to investigate the pharmacology of basophil activation. Testing other basophil agonists and deepening the knowledge of the transduction pathways and of gene expression may substantiate these views.

## Competing interests

The authors declare that they have no competing interests.

## Authors' contributions

SC and PB conceived the study, performed the experiments and wrote the manuscript, AV and RO assisted in the flow cytometry setting and handling, MDG and PS participated in the subjects recruitment and in blood specimen selection and GT participated in study design and coordination. All authors read and approved the final manuscript.

## Supplementary Material

Additional file 1 Mean of fluorescence intensity (MFI) of basophil markers in resting and agonist stimulated human basophils. The data provided represent the statistical analysis of various membrane markers of human basophils. Click here for file

## References

[B1] Boumiza R, Debard AL, Monneret G (2005). The basophil activation test by flow cytometry: recent developments in clinical studies, standardization and emerging perspectives. Clin Mol Allergy.

[B2] Triggiani M, Marone G (2006). Basophil's secrets revealed by flow cytometry. Allergy.

[B3] Ebo DG, Sainte-Laudy J, Bridts CH, Mertens CH, Hagendorens MM, Schuerwegh AJ, De Clerck LS, Stevens WJ (2006). Flow-assisted allergy diagnosis: current applications and future perspectives. Allergy.

[B4] Abuaf N, Rostane H, Rajoely B, Gaouar H, Autegarden JE, Leynadier F, Girot R (2008). Comparison of two basophil activation markers CD63 and CD203c in the diagnosis of amoxicillin allergy. Clin Exp All.

[B5] de Week AL, Sanz ML, Gamboa PM, Aberer W, Bienvenu J, Bianca M, Demoly P, Ebo DG, Mayorga L, Monneret G, Sainte-Laudy J (2008). Diagnostic tests based on human basophils: more potentials and perspectives than pitfalls: II Technical issues. J Investig Allergol Clin Immunol.

[B6] Szegedi A, Irinyi B, Gal M, Hunyadi J, Danko K, Kiss E, Sipka S, Szegedi G, Gyimesi E (2006). Significant correlation between the CD63 assay and the histamine release assay in chronic urticaria. Br J Dermatol.

[B7] Sudheer PS, Hall JE, Read GF, Rowbottom AW, Williams PE (2005). Flow cytometric investigation of peri-anaesthetic anaphylaxis using CD63 and CD203c. Anaesthesia.

[B8] Yasnowsky KM, Dreskin SC, Efaw B, Schoen D, Vedanthan PK, Alam R, Harbeck RJ (2006). Chronic urticaria sera increase basophil CD203c expression. J Allergy Clin Immunol.

[B9] Vasagar K, Vonakis BM, Gober LM, Viksman A, Gibbons SP, Saini SS (2006). Evidence of in vivo basophil activation in chronic idiopathic urticaria. Clin Exp Allergy.

[B10] Ebo DG, Hagendorens MM, Schuerwegh AJ, Beirens LM, Bridts CH, De Clerck LS, Stevens WJ (2007). Flow-assisted quantification of in vitro activated basophils in the diagnosis of wasp venom allergy and follow-up of wasp venom immunotherapy. Cytometry B Clin Cytom.

[B11] Kleine-Tebbe J, Erdmann S, Knol EF, Macglashan DW, Poulsen LK, Gibbs BF (2006). Diagnostic tests based on human basophils: potentials, pitfalls and perspectives. Int Arch Allergy Immunol.

[B12] de Weck AL, Sanz ML, Gamboa PM, Aberer W, Bienvenu J, Bianca M, Demoly P, Ebo DG, Mayorga L, Monneret G, Saint-Laudy J (2008). Diagnostic tests based on human basophils: more potentials and perspectives than pitfalls. Int J All Immunol.

[B13] Monneret G (2008). Is the time for CRTH2/DP2 in a flow cytometric basophil activation test?. Clin Exp All.

[B14] Hennersdorf F, Florian S, Jakob A, Baumgartner K, Sonneck K, Nordheim A, Biedermann T, Valent P, Buhring HJ (2005). Identification of CD13, CD107a, and CD164 as novel basophil-activation markers and dissection of two response patterns in time kinetics of ige-dependent upregulation. Cell Res.

[B15] Zola H, Swart B, Banham A, Barry S, Beare A, Bensussan A, Boumsell L, Buckley CD, Buhring HJ (2007). CD molecules 2006 – Human cell differentiation molecules. J Immunol Methods.

[B16] Florian S, Sonneck K, Czerny M, Hennersdorf F, Hauswirth AW, Buhring HJ, Valent P (2006). Detection of novel leukocyte differentiation antigens on basophils and mast cells by HLDA8 antibodies. Allergy.

[B17] Ocmant A, Peignois Y, Mulier S, Hanssens L, Michils A, Schandene L (2007). Flow cytometry for basophil activation markers: The measurement of CD203c up-regulation is as reliable as CD63 expression in the diagnosis of cat allergy. J Immunol Methods.

[B18] Ducrest S, Meier F, Tschopp C, Pavlovic R, Dahinden CA (2005). Flowcytometric analysis of basophil counts in human blood and inaccuracy of hematology analyzers. Allergy.

[B19] Ebo DG, Lechkar B, Schuerwegh AJ, Bridts CH, De Clerck LS, Stevens WJ (2002). Validation of a two-color flow cytometric assay detecting in vitro basophil activation for the diagnosis of IgE-mediated natural rubber latex allergy. Allergy.

[B20] Sainte-Laudy J, Boumediene A, Touraine F, Orsel I, Brianchon C, Bonnaud F, Cogne M (2007). Use of both CD63 up regulation and IgE down regulation for the flow cytometric analysis of allergen induced basophil activation. Definition of an activation index. Inflamm Res.

[B21] Petrausch U, Haley D, Miller W, Floyd K, Urba WJ, Walker E (2007). Polychromatic flow cytometry: a rapid method for the reduction and analysis of complex multiparameter data. Cytometry.

[B22] Bigos M, Baumgarth N, Jager JC, Herman OC, Nozaki T, Stovel RT, Parks DR, Herzenberg LA (1999). Nine colours eleven parameter immunophenotyping using three laser flow cytometry. Cytometry.

[B23] Gane P, Pecquet C, Lambin P, Abuaf N, Leynadier F, Rouger P (1993). Flow cytometric evaluation of human basophils. Cytometry.

[B24] Han X, Jorgensen JL, Brahmandam A, Schlette E, Huh YO, Shi Y, Awagu S, Chen W (2008). Immunophenotypic study of basophils by multiparameter flow cytometry. Arch Pathol Lab Med.

[B25] Buhring HJ, Streble A, Valent P (2004). The basophil-specific ectoenzyme E-NPP3 (CD203c) as a marker for cell activation and allergy diagnosis. Int Arch Allergy Immunol.

[B26] Kinet JP (1999). The high-affinity IgE receptor (FcepsilonRI): from physiology to pathology. Annu Rev Immunol.

[B27] Boumiza R, Monneret G, Forissier MF, Savoye J, Gutowski MC, Powell WS, Bienvenu J (2003). Marked improvement of the basophil activation test by detecting CD203c instead of CD63. Clin Exp Allergy.

[B28] Harris N, Jou JM, Devoto G, Lotz J, Pappas J, Wranovics D, Wilkinson M, Fletcher SR, Kratz A (2005). Performance evaluation of the ADVIA 2120 hematology analyzer: an international multicenter clinical trial. Lab Hematol.

[B29] Knol EF, Kuijpers TW, Mul FP, Roos D (1993). Stimulation of human basophils results in homotypic aggregation. A response independent of degranulation. J Immunol.

[B30] Maecker HT, Frey T, Nomura LE, Trotter J (2004). Selecting fluorochrome conjugates for maximum sensitivity. Cytometry.

[B31] Tsang S, Hayashi M, Zheng X, Campbell A, Schellenberg R (2000). Simplified purification of human basophils. J Immunol Methods.

[B32] Varro R, Chen CH (2000). A no-wash 3-color basophil degranulation flow assay using the CD123+HLA-DR-phenotype for basophil classification. Cytometry.

[B33] Sanz ML, Maselli JP, Gamboa PM, Oehling A, Dieguez I, de Weck AL (2002). Flow cytometric basophil activation test: a review. J Investig Allergol Clin Immunol.

[B34] Knol EF, Mul FP, Jansen H, Calafat J, Roos D (1991). Monitoring human basophil activation via CD63 monoclonal antibody 435. J Allergy Clin Immunol.

[B35] Platz IJ, Binder M, Marxer A, Lischka G, Valent P, Buhring HJ (2001). Hymenoptera-venom-induced upregulation of the basophil activation marker ecto-nucleotide pyrophosphatase/phosphodiesterase 3 in sensitized individuals. Int Arch Allergy Immunol.

[B36] Binder M, Fierlbeck G, King T, Valent P, Buhring H (2002). Individual hymenoptera venom compounds induce upregulation of the basophil activation marker ectonucleotide pyrophosphatase/phosphodiesterase 3 (CD203c) in sensitized patients. Int Arch Allergy Immunol.

[B37] Eberlein-Konig B, Schmidt-Leidescher C, Rakoski J, Behrendt H, Ring J (2006). In vitro basophil activation using CD63 expression in patients with bee and wasp venom allergy. J Investig Allergol Clin Immunol.

[B38] Sanz ML, Garcia-Aviles MC, Tabar AI, Anda M, Garcia BE, Barber D, Salcedo G, Rihs HP, Raulf-Heimsoth M (2006). Basophil Activation Test and specific IgE measurements using a panel of recombinant natural rubber latex allergens to determine the latex allergen sensitization profile in children. Pediatr Allergy Immunol.

[B39] Gane P, Pecquet C, Crespeau H, Lambin P, Leynadier F, Rouger P (1995). Flow cytometric monitoring of allergen induced basophil activation. Cytometry.

[B40] Yoshimura C, Yamaguchi M, Iikura M, Izumi S, Kudo K, Nagase H, Ishii A, Walls AF, Ra C, Iwata T, Igarashi T, Yamamoto K, Hirai K (2002). Activation markers of human basophils: CD69 expression is strongly and preferentially induced by IL-3. J Allergy Clin Immunol.

[B41] Youssef LA, Schuyler M, Gilmartin L, Pickett G, Bard JD, Tarleton CA, Archibeque T, Qualls C, Wilson BS, Oliver JM (2007). Histamine release from the basophils of control and asthmatic subjects and a comparison of gene expression between "releaser" and "nonreleaser" basophils. J Immunol.

[B42] Furstenau CR, Trentin Dda S, Barreto-Chaves ML, Sarkis JJ (2006). Ecto-nucleotide pyrophosphatase/phosphodiesterase as part of a multiple system for nucleotide hydrolysis by platelets from rats: kinetic characterization and biochemical properties. Platelets.

[B43] Goding JW, Grobben B, Slegers H (2003). Physiological and pathophysiological functions of the ecto-nucleotide pyrophosphatase/phosphodiesterase family. Biochim Biophys Acta.

[B44] Terkeltaub R (2006). Physiological and pathologic functions of the NPP nucleotide pyrophosphatase/phosphodiesterase family focusing on NPP1 in calcification. Purinergic Signalling.

[B45] Bellavite P, Chirumbolo S, Lippi G, Guzzo P, Santonastaso C (1993). Homologous priming in chemotactic peptide stimulated neutrophils. Cell Biochem Funct.

[B46] Bellavite P, Carletto A, Biasi D, Caramaschi P, Poli F, Suttora F, Bambara LM (1994). Studies of skin-window exudate human neutrophils: complex patterns of adherence to serum coated surfaces in dependence of fMLP doses. Inflammation.

[B47] Youssef LA, Schuyler M, Gilmartin L, Pickett G, Bard JDJ, Tarleton CA, Archibeque T, Qualls C, Wilson BS, Oliver JM (2007). Histamine release from the basophils of control and asthmatic subjects and a comparison of gene expression between "releaser" and "nonreleaser" basophils. J Immunol.

[B48] Chirumbolo S, Ortolani R, Vella A, Tridente G, Bellavite P (2008). A six-color polychromatic flow cytometry to evaluate human basophil activation. Cytometry.

