# Supplementary material for: Differential response of human basophil activation markers: a multi-parameter flow cytometry approach
Source: Clin Mol Allergy. 2008 Oct 16;6:12. doi: 10.1186/1476-7961-6-12 (PMC2584049; doi:10.1186/1476-7961-6-12)
Supplement: Additional file 1 — Mean of fluorescence intensity (MFI) of basophil markers in resting and agonist stimulated human basophils. The data provided represent the statistical analysis of various membrane markers of human basophils. [file 1476-7961-6-12-S1.doc]

**Table 1 – Mean of fluorescence intensity (MFI) of basophil markers in resting and agonist stimulated human basophils**

| MARKERS | RESTING | | | | | fMLP 10^-7^ M | | | | | Anti-IgE 1 μg/ml | | | | |
| --- | --- | --- | --- | --- | --- | --- | --- | --- | --- | --- | --- | --- | --- | --- | --- |
|  | MFI | SD | Min | Max | N | MFI | SD | Min | Max | N | MFI | SD | Min | Max | N |
| CD45-APCCy7 | **2861.12** | 785.6 | 1908 | 4067 | 12 | **7126.21** | 3481.8 | 1760 | 12313 | 14 | **4689.50** | 3212.3 | 1214 | 8654 | 6 |
| CD123-PECy5 | **28425.45** | 12044.4 | 14709 | 47947 | 18 | **27850.08** | 9810.8 | 14750 | 46706 | 18 | **28872.57** | 8396.5 | 16000 | 37543 | 6 |
| HLADR-PECy7 | **223.64** | 87.2 | 127 | 409 | 18 | **239.00** | 86.1 | 132 | 407 | 18 | **210.86** | 102.2 | 132 | 502 | 6 |
| CD63-FITC | **619.76** | 383.2 | 207 | 1454 | 20 | **14980.74** | 12130.6 | 3957 | 47786 | 20 | **3004.50** | 2594.6 | 770 | 5673 | 6 |
| CD63-FITC (a)  % bright cells | **3.12** | 2.5 | 0.2 | 9.2 | 19 | **33.44** | 11.9 | 10.4 | 52.9 | 20 | **13.67** | 7.6 | 7.3 | 22.4 | 4 |
| CD203c-PE | **2112.05** | 1602.8 | 993 | 5263 | 19 | **6508.74** | 2709.5 | 2955 | 13403 | 19 | **3904.80** | 3547.0 | 2109 | 8235 | 4 |
| CD13-APC | **3056.65** | 2150.7 | 887 | 9435 | 17 | **6216.31** | 3731.7 | 2766 | 13566 | 14 | **2148.25** | 1894.9 | 1753 | 4596 | 3 |
| CD69-APC | **863.33** | 204.0 | 629 | 1001 | 4 | **1316** | 274.4 | 1111 | 1521 | 4 | **1362** | 300.5 | 1061 | 1662 | 3 |

(a) Reported values are not MFI but percentage of CD63 expressing (up-regulated) cells as respect to the whole population
